# Synergistic activity of the mTOR inhibitor ridaforolimus and the antiandrogen bicalutamide in prostate cancer models

**DOI:** 10.3892/ijo.2012.1487

**Published:** 2012-05-18

**Authors:** RACHEL M. SQUILLACE, DAVID MILLER, SCOTT D. WARDWELL, FRANK WANG, TIM CLACKSON, VICTOR M. RIVERA

**Affiliations:** 1 Departments of Biology and; 2 Pharmacology, ARIAD Pharmaceuticals Inc., Cambridge, MA, USA

**Keywords:** ridaforolimus (AP23573; MK-8669), bicalutamide, prostate cancer, mTOR inhibition, androgen receptor, combination therapy

## Abstract

Although androgen ablation therapy is the foundation of current prostate cancer treatment, most patients ultimately develop castration-resistant disease. One proposed mechanism to account for androgen receptor (AR) activity in the castrate environment is via crosstalk with other signaling pathways. Specifically, reciprocal interactions between the AKT/mTOR and AR pathways have been implicated in prostate cancer progression. Here, we used the potent inhibitor ridaforolimus to target mTOR signaling alone and in combination with AR blockade by bicalutamide to examine the effect of abrogating these signaling pathways. Ridaforolimus treatment inhibited the proliferation of all six prostate cancer cell lines examined with the greatest sensitivity associated with loss of PTEN and elevated AKT/mTOR pathway activity. Dual inhibition of the AR and mTOR signaling pathways provided further benefit with the ridaforolimus-bicalutamide combination producing synergistic antiproliferative effects in prostate cancer cells *in vitro* when compared with each agent alone. Pharmacodynamic analysis confirmed that combination treatment resulted in full inhibition of each of the respective pathways. Importantly, the ridaforolimus-bicalutamide combination exhibited potent anti-tumor activity with parallel reductions in plasma PSA levels *in vivo*. Taken together, ridaforolimus exhibited potent antiproliferative and antitumor activity in prostate cancer models and the addition of bicalutamide represents a potentially effective combination strategy for patient therapy.

## Introduction

Prostate cancer is the most frequently diagnosed malignancy and second leading cause of cancer death in males both in the United States and United Kingdom. A unique characteristic of these tumors is that they are exquisitely dependent on androgen for development, growth and survival (1–3). Hence, in addition to their normal physiological roles in the growth and development of male sex organs, androgens play an equally critical function in the abnormal growth of prostate cancer. In both contexts, these effects are mediated through activation of the androgen receptor (AR) signaling pathway (4). Upon diagnosis patients are typically subjected to androgen ablation therapy involving either surgical or chemical castration. This latter process is achieved through the use of selective antiandrogen agents, such as gonadotropin-releasing hormone (GnRH) agonists or second generation AR inhibitors like bicalutamide (Casodex). Unfortunately, while blockade of AR is initially effective in achieving clinically relevant remissions, most patients relapse and progress with castration-resistant disease within 12–24 months (5). Previously considered to be androgen-independent, it is now emerging that these recurrent prostate cancers may still rely on AR signaling for growth and survival. The mechanisms by which these more aggressive tumors retain AR activity and AR-mediated gene expression are still unclear, although it has been hypothesized that the development of intratumoral steroidogenesis may contribute to castration-resistant tumor growth (6). First-line treatment for castration-resistant prostate cancer typically consists of docetaxel in combination with prednisone or estramustine (7). However, no consensus exists regarding the best approach following docetaxel failure (8). Second-line approaches including hormone therapy, taxane or immunotherapy often fail to dramatically impact patient survival. A unifying feature of advanced or metastatic disease therefore is that patient prognosis is dismal as therapeutic options become more limited.

The AR receptor pathway mediates the transcriptional regulation of multiple genes in prostate cancer, including those that promote tumor cell survival and proliferation. In addition, the ability of AR to crosstalk with other key growth factor signaling pathways in prostate cancer has been established (9). In particular, recent studies have identified several mechanisms for regulation of AR by the mammalian target of rapamycin (mTOR) signaling cascade and vice versa (10–12). mTOR is a serine/threonine kinase downstream of PI3K/AKT that acts as a checkpoint for both cellular nutritional status and cell cycle control (13–15). Of note, dysregulation of the PI3K/AKT/mTOR pathway has been implicated in the malignant transformation accompanying prostate cancer progression (16). Moreover, loss-of-function of the tumor suppressor gene PTEN, which results in constitutive activation of AKT and upregulation of mTOR activity, has been implicated in the etiology of numerous human cancers including more than 50% of advanced prostate tumors (17–19). Further, it has been demonstrated that tumors that harbor deletions or defects in PTEN are, in general, hypersensitive to inhibition of mTOR (20–22). This provides a clear biological rationale for the blockade of mTOR activity as a potential therapeutic point of intervention for prostate cancer.

Ridaforolimus (AP23573, MK-8669), a non-prodrug analog of rapamycin, is a potent and selective inhibitor of mTOR (23) currently under clinical investigation as a targeted cancer. Interestingly, ridaforolimus has shown promising single-agent activity in a phase II trial of advanced, progressive endometrial cancer (24). Similar to prostate cancer, this tumor type is also characterized by a high incidence of functional inactivation of PTEN (25–27). In preclinical models of endometrial cancer, we previously demonstrated an association between PTEN loss and ridaforolimus sensitivity (28). A number of reports have also suggested targeting mTOR in prostate cancer (29–31). Here we investigated both the effects of mTOR inhibition alone, as well as in combination with AR blockade, in models of prostate cancer. We show that simultaneous treatment with ridaforolimus and bicalutamide results in synergistic antiproliferative effects *in vitro* and *in vivo*. These findings support the potential therapeutic value of dual inhibition of the AR and mTOR signaling pathways as a valid approach for the treatment of patients with this disease.

## Materials and methods

### Cell lines and reagents

All cell lines used in this study were obtained from the American Type Culture Collection with the exception of the C4-2 line, which was a kind gift from Dr George Thalmann (University of Bern, Switzerland). Cells were maintained and cultured according to standard techniques at 37°C in 5% (v/v) CO2 using culture medium recommended by the supplier. Ridaforolimus (AP23573; MK-8669) was synthesized at ARIAD Pharmaceuticals and prepared in ethanol to a 1 mM stock concentration. For *in vitro* cellular assays ridaforolimus was diluted in the optimal medium. For *in vivo* experiments, ridaforolimus was diluted in a vehicle of 4% ethanol, 5% Tween 80, and 5% propylene glycol. Bicalutamide (Casodex) was purchased from Zheijang Esun Chemical Co., Ltd. (China).

### In vitro proliferation assays

Proliferation assays were performed as previously described (23). Briefly, exponentially growing cell lines were plated into two 96-well plates and incubated overnight at 37°C. Twenty-four hours later one plate was aspirated and stored at −80°C and the other treated with 10-fold serial concentrations of ridaforolimus (1000 nM to 0.0001 nM) or vehicle (ethanol). Following 72 h culture at 37°C, the plates were assessed simultaneously for cell growth using the CyQUANT Cell Proliferation Assay kit (Invitrogen). The parameters measured were: Doubling time (DT) = [0.301*(72)/LOG(Day4/Day1)]; Doublings = 72/DT; and Cell growth rate (%) = Doublings ridaforolimus /Doublings vehicle *100. The maximal inhibitory effect (I_max_) measure was used to determine relative sensitivity of each cell line. I_max_ = 100 – cell growth rate (%) at the dose whereby maximum inhibition is observed. Compound combination proliferation assays were performed similarly except cell growth was determined as the change in cell number between vehicle control and compound treated cells after 72 h in culture. The average (± SD) of n ≥3 individual experiments are reported.

### Median effect analysis

Cells were seeded as described for the *in vitro* proliferation assay and combination treatments of ridaforolimus and bicalutamide were performed with fixed 1:1 ratios of concentrations that induced half the maximal effect (i.e. EC_50_ values) for each drug. Two-fold serial dilutions above and below the EC_50_ values were added to the cell cultures for 72 h. The nature of the ridaforolimus-bicalutamide combination interaction was evaluated using the combination index (CI) method of Chou and Talalay (32) and values were generated using Median Effect analysis (CalcuSyn Software; Biosoft).

### Anchorage-independent cell proliferation analysis

C4-2 cells were immobilized in 6-well dishes in culture medium containing 0.3% agarose. The soft agar layer containing the cells was then overlaid with a liquid media layer containing one of the following: 0.5 nM ridaforolimus, 10 *μ*M bicalutamide, 0.5 nM ridaforolimus + 10 *μ*M bicalutamide, or media alone (no treatment). The cells were cultured at 37°C for 2 weeks, with the liquid media layer being replaced with fresh media/drug treatment every three to four days. Colonies were then counted using a Universal Hood II Molecular Imager^®^ ChemiDoc™XRS System and Quantity One^®^ SW 1-D Analysis software (Bio-Rad Laboratories, Inc.). The relative colony formation for each treatment group was then calculated as a percentage of the untreated group using the formula: (# colonies treatment group/ # colonies untreated group)*100. The p-value was calculated using the Student’s t-test.

### Flow cytometric analysis

C4-2 cells cultured in 10 cm dishes were treated for 24 h with one of the following: 50 nM ridaforolimus, 50 *μ*M bicalutamide, 50 nM ridaforolimus + 50 *μ*M bicalutamide, or vehicle control. The cells were then harvested and fixed with 70% ethanol / 30% PBS overnight at 4°C. Fixed cells were washed and then sequentially incubated with 50 *μ*g/ml RNase A (37°C for 30 min) and 20 *μ*g/ml propidium iodide (room temperature for 30 min in the dark). DNA content was analyzed using a FACSort flow cytometer and CellQuest V3.1 software (Becton-Dickinson and Company). The percentage of cells in each phase of the cell cycle was then estimated from the FL2-A channel data using ModFit LT for Mac V2.0 software (Verity Software House, Inc.). The p-value was calculated using the Student’s t-test.

### Biomarker expression and in vitro pharmacodynamics

Cell lines were harvested during exponential growth phase and immunoblotted for markers of the AR and AKT/mTOR pathways. Lysates were loaded left to right according to decreasing level of ridaforolimus sensitivity determined by I_max_. For pharmacodynamic analysis, cells were treated with 0.5 nM ridaforolimus and/or 10 *μ*M bicalutamide then harvested and assessed by immunoblotting for PTEN, AKT, p-AKT (Ser^473^), S6, p-S6 (Ser^235/236^), p-4E-BP1 (Ser^65^/Thr^70^) and VDAC (loading control) expression (Cell Signaling Technology). PTEN status of cell lines was determined using the Sanger Cancer Genome Project mutation database (http://www.sanger.ac.uk/genetics/CGP) and confirmed by immunoblotting.

### PSA ELISA

Cells were treated for 72 h with single agent or combination compound treatment as described for the *in vitro* proliferation assay, supernatants were harvested, and PSA Quantikine Immunoassay was performed according to the manufacturers protocols (R&D Systems).

### Subcutaneous tumor model

Prostate cancer xenografts were established by the subcutaneous implantation of C4-2 cells (5×10^6^ cells + matrigel) at the right flank area of six to eight week old male nude mice (nu/nu strain) (Charles River Laboratories; Wilmington, MA). For analysis of efficacy, when the average tumor volume reached approximately 200 mm^3^ mice were administered the indicated dose of ridaforolimus i.p. (n=10 mice/condition) daily for 5 days followed by a 2 day break, or bicalutamide p.o. daily, or the combination. Three cycles of dosing were completed (21 days). Mean tumor volume volumes were calculated for each treatment group by caliper measurements using the following formula: tumor volume = (length × width^2^)/2. Blood was harvested from mice on days 0, 7, 14 and 21 for PSA ELISA. Differences between the multiple treatment groups were analyzed by one-way ANOVA test.

### Ethical treatment of animals

All of the animal experiments were conducted in strict accordance with the National Institute of Health Guide for the Care and Use of Laboratory Animals. The protocol was approved by the Institutional Animal Care and Use Committees, ARIAD Pharmaceuticals, Inc. (Protocol Number: 08-01). All efforts were made to minimize suffering.

## Results

### Loss of PTEN and elevated AKT/mTOR activity are associated with ridaforolimus sensitivity in prostate cell lines

**Figure 1:**
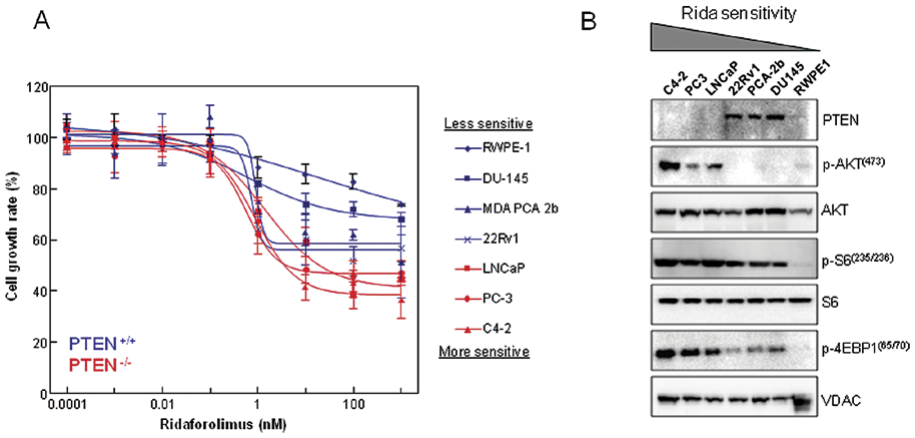
PTEN loss and elevated AKT/mTOR activity is associated with ridaforolimus sensitivity in prostate cell lines. To evaluate the level of sensitivity of cells to ridaforolimus we measured the effect on the rate of cell proliferation, rather than on the absolute cell number since the effect of a cytostatic drug on absolute cell number is directly influenced by the intrinsic cell doubling time. (A) Determination of ridaforolimus sensitivity in a panel of prostate cancer as well as prostate epithelial (RWPE-1) cell lines cultured with ridaforolimus over a 0.0001–1,000 nM concentration range for 3 days. PTEN wild-type cells (PTEN^+/+^) are shown in blue and PTEN null (PTEN^−/−^) shown in red. (B) Relationship between PTEN/AKT/mTOR pathway markers and ridaforolimus sensitivity. Cell lines are presented with decreasing sensitivity to ridaforolimus, as determined in (A). Cellular extracts from the prostate cell lines were prepared and equal amounts of total protein were analyzed by immunoblotting for PTEN, phospho-AKT (Ser^473^), AKT, phospho-S6 (Ser^235/236^), ribosomal protein S6, phospho-4EBP1 (Ser^65^/Thr^70^) and VDAC (as a loading control).

We examined the effect that single agent ridaforolimus treatment had on cell proliferation using a series of prostate-derived cell lines (Fig. 1A). Exposure to nanomolar concentrations of ridaforolimus reduced cellular proliferation in each of the 6 prostate cancer cell lines (DU-145, MDA PCA 2b, 22Rv1, LNCaP, PC-3 and C4-2) with maximal inhibition (I_max_) ranging from 20–60%. In contrast, ridaforolimus was least effective in inhibiting the cell growth rate of the immortalized normal prostatic epithelial line RWPE-1. Notably, cellular PTEN status was associated with drug sensitivity, as the PTEN-null cancer cell lines showed the greatest degree of inhibition. Loss of PTEN typically leads to constitutive activation of downstream components of the PI3K pathway, including the AKT and mTOR kinases. As confirmation, we examined the expression levels and activation state of this signaling cascade and compared that to ridaforolimus sensitivity in our panel of prostate lines (Fig. 1B). As expected, there was a concomitant increase in phospho-AKT (Ser^473^) levels observed in the PTEN^−/−^ cell lines (LNCaP, PC-3, C4-2). These same three lines also demonstrated hyperactivation of the mTOR pathway, as evidenced by elevated phosphorylation of the key downstream targets ribosomal protein S6 and 4E-BP1 (Fig. 1B). Together, these data indicate that PTEN loss and aberrant mTOR signaling are intrinsic cellular properties associated with ridaforolimus sensitivity in prostate cancer lines.

### Simultaneous blockade of both AR and mTOR pathways in cancer, but not normal prostate lines, results in synergistic growth inhibition

**Figure 2:**
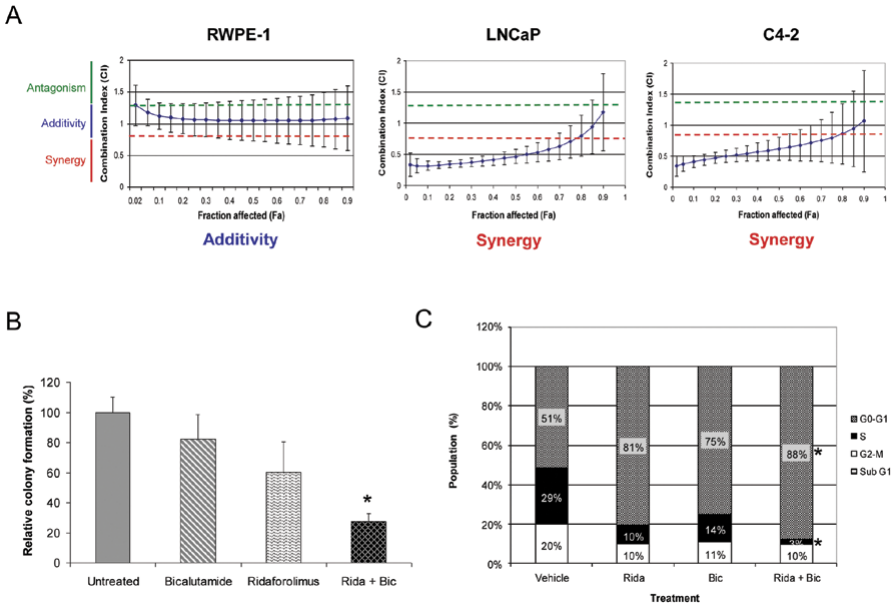
Simultaneous AR and mTOR pathway blockade results in synergistic growth inhibition in prostate cancer lines. (A) RWPE-1, LNCaP and C4-2 cell lines were treated with increasing concentrations of ridaforolimus, bicalutamide, or both, and the effects on proliferation determined. The Combination Index (CI) was calculated using Median Effect analysis. Strict criteria were applied to drug interaction analysis, where synergy was defined as CI <0.75, additivity as >0.75 CI <1.25, and antagonism as CI >1.25. Data expressed as mean CI (± SD), determined for a range of drug concentrations and a fractional effect (Fa) of 0.2 to 0.8 over the complete dosing range. (B) Soft agar clonogenic assay to determine the effects of the ridaforolimus and bicalutamide combination on anchorage-independent growth of C4-2 prostate cancer cells. C4-2 cells were treated with medium alone, bicalutamide (10 *μ*M), ridaforolimus (0.5 nM) or the combination (Rida + Bic) for 2 weeks. The percentage colony formation (compared to untreated controls) are presented as means ± SD for duplicate experiments. ^*^p-value ≤0.01 compared with vehicle treated cells. (C) Ridaforolimus and bicalutamide in combination induce cell cycle arrest in prostate cancer cells. C4-2 prostate cancer cells were treated with vehicle, ridaforolimus (Rida; 50 nM), bicalutamide (Bic; 50 *μ*M) or the combination (Rida + Bic) for 24 h. Cells were harvested, stained with propidium iodide and analyzed by flow cytometry to determine DNA content. The percentage of cells in G1, S or G2/M phase was calculated from FL-2 histograms using ModFit Lt software. ^*^p-value ≤0.05 compared with single agents or vehicle treated cells.

Loss of PTEN and consequent elevation of AKT activity can promote both mTOR as well as AR-dependent proliferation (33). Further, it has been suggested that one function of AR in PTEN-deficient prostate cancer cells is to promote the pathologic activation of mTOR (11), providing a potential mechanistic link between these two pathways in prostate cancer. Based on these considerations, we evaluated the combinatorial effects of ridaforolimus and the antiandrogen bicalutamide in inhibiting the growth of prostate cell lines. To examine this, the normal prostate PTEN wild-type line, RWPE-1, and two PTEN^−/−^ tumor lines, LNCaP and C4-2, were treated *in vitro* with a combination of ridaforolimus and bicalutamide. LNCaP is a well characterized, androgen-dependent cell line that represents the early stages of prostate cancer progression. The metastatically derived subline of LNCaP, C4-2, has been traditionally considered androgen-independent. Indeed, we have found that C4-2 can grow in the absence of androgens *in vitro* and in castrated mice, although proliferation in both model systems is substantially reduced (data not shown). However, they do respond to androgen and are sensitive to bicalutamide in an androgen-rich environment (see below). Therefore, C4-2 cells are a more progressed form of prostate cancer compared with LNCaP, but they are not fully androgen-independent. Combinatorial activity was determined using the Median Effect method to establish whether the combinations exhibited antagonistic, additive or synergistic activity (Fig. 2A). RWPE-1 cells displayed only a simple additive effect when treated with these two agents together. In stark contrast, the combination was found to be strongly synergistic in both prostate cancer cell lines. The combinatorial benefit was further demonstrated by significant inhibition of anchorage-independent growth in the C4-2 cell line (Fig. 2B). These findings suggest the potential for combining therapy in prostate cancer patients with inhibitors of both AR and mTOR pathways.

### Ridaforolimus and bicalutamide combination treatment promotes cell cycle arrest in prostate cancer cells

Ridaforolimus exerts its antiproliferative effects on cancer cells through primarily cytostatic, not cytotoxic, activities (23). We thus investigated the mode of action of the ridaforolimusbicalutamide combination on cell cycle progression and survival of C4-2 cells. Single agent treatment alone led to a decrease in both S and G2/M phases with a concomitant accumulation in the G1 phase of the cell cycle (Fig. 2C). Consistent with the enhanced growth inhibition of these cells shown in Fig. 2A, the effect of combination treatment was more pronounced, resulting in an almost complete G1 arrest in this cell line. Indeed, both the increase in the proportion of cells in G1 phase and reduction in S phase were significantly different from either vehicle controls or each individual agent alone. No increase in the sub-G1 fraction was observed with any treatment indicating no significant pro-apoptotic activity. This result was confirmed by additional studies which failed to detect an increase in other apoptotic markers including cleaved PARP and caspase-3 (data not shown).

### PSA levels mirror cell growth of ridaforolimus-bicalutamide treated prostate cancer lines

**Figure 3:**
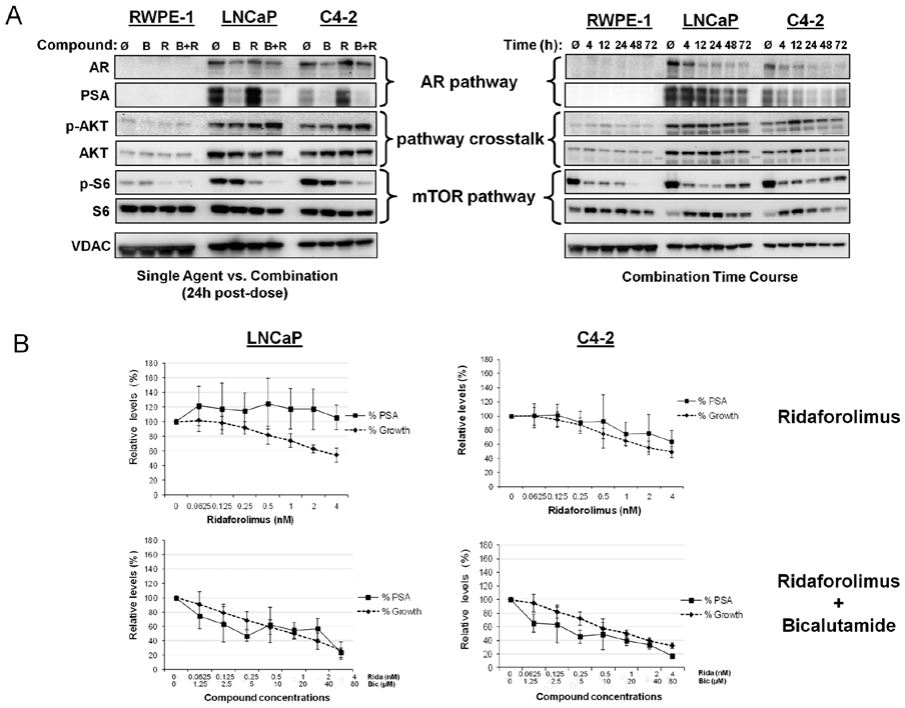
Combination of ridaforolimus plus bicalutamide inhibits AR and mTOR signaling; PSA levels mirror cell growth in combination-treated prostate cell lines. (A) In the left panel RWPE-1, LNCaP and C4-2 cells were treated for 24 h with vehicle alone (Ǿ), 10 *μ*M bicalutamide (B), 0.5 nM ridaforolimus (R), or the combination (B+R). In the right panel, RWPE-1, LNCaP and C4-2 cells were treated with the combination of ridaforolimus and bicalutamide for up to 3 days with lysates harvested at the indicated times. Cellular extracts were immunoblotted for AR, PSA, phospho-S6 (Ser^235/236^), ribosomal protein S6 and VDAC (as a control). (B) LNCaP and C4-2 cells were treated with ridaforolimus as a single agent at the indicated concentrations (upper panels), or with the ridaforolimus/bicalutamide combination at the range of concentrations indicated (lower panels) for 3 days. PSA levels in the supernatant were determined by ELISA and relative secretion presented as the ratio of compound- versus vehicle-treated cells. The effects on proliferation were evaluated and cell growth shown as a percentage of vehicle controls. Data presented as means of ≥3 individual experiments ± SD.

mTOR blockade can result in increased AR transcriptional activity and consequent PSA expression, independent of effects on cell growth (12). This finding has important clinical implications, as plasma PSA levels in prostate cancer patients are used as a measure of tumor growth and disease progression. As shown in Fig. 3A (left panel), the immortalized RWPE-1 cell line did not exhibit elevation of AR or PSA expression, as expected. Bicalutamide treatment resulted in significant suppression of PSA levels and moderate decrease of AR expression in both LNCaP and C4-2 prostate cancer lines. In contrast, addition of ridaforolimus to the androgen-dependent LNCaP line resulted in an increase in PSA expression and a similar response was observed in the C4-2 cells. However, in both cases, the combination of ridaforolimus and bicalutamide abrogated this effect. Indeed, combination treatment resulted in the temporal inhibition of both PSA and AR expression (Fig. 3A, right panel) thus, bicalutamide not only inhibited basal levels of AR transcriptional activity, but was sufficient to block the ridaforolimus-induced stimulation of PSA. Ridaforolimus alone and in combination promoted a modest increase of p-AKT levels in both cancer lines at the 24 h time point, however, this effect was not sustained with the combination over the longer 72 h time course. As expected, single agent ridaforolimus reduced phospho-S6 levels in the tumor lines. Interestingly, the addition of bicalutamide also potentiated this effect, providing further evidence of pathway crosstalk in these cancer cells.

Having determined that mTOR inhibition by ridaforolimus leads to increased PSA expression, we next examined whether this effect related to changes in cell growth. To investigate this, cell lines were treated with either ridaforolimus alone or bicalutamide and the relative levels of cellular proliferation and PSA secretion compared (Fig. 3B). As expected, RWPE-1 cells, which lack AR signaling and exhibit low endogenous mTOR activity, did not secrete PSA (data not shown). In LNCaP cells, despite a dose-dependent decrease in cell proliferation following ridaforolimus treatment, the levels of secreted PSA did not change. Combination treatment, however, did result in a parallel effect on both PSA secretion and cell growth (Fig. 3B). In C4-2 cells, a decrease in cell growth with ridaforolimus treatment was accompanied by a slight decrease in PSA levels at the highest doses tested. Similar to the LNCaP line, a marked reduction in PSA levels mirrored growth inhibition after addition of the ridaforolimus-bicalutamide combination. Taken together, these data support the utility of PSA secretion as a readout of cell proliferation in combination treated cancer lines *in vitro.*

### Ridaforolimus plus bicalutamide inhibits prostate tumor growth and reduces plasma PSA level in vivo

**Figure 4:**
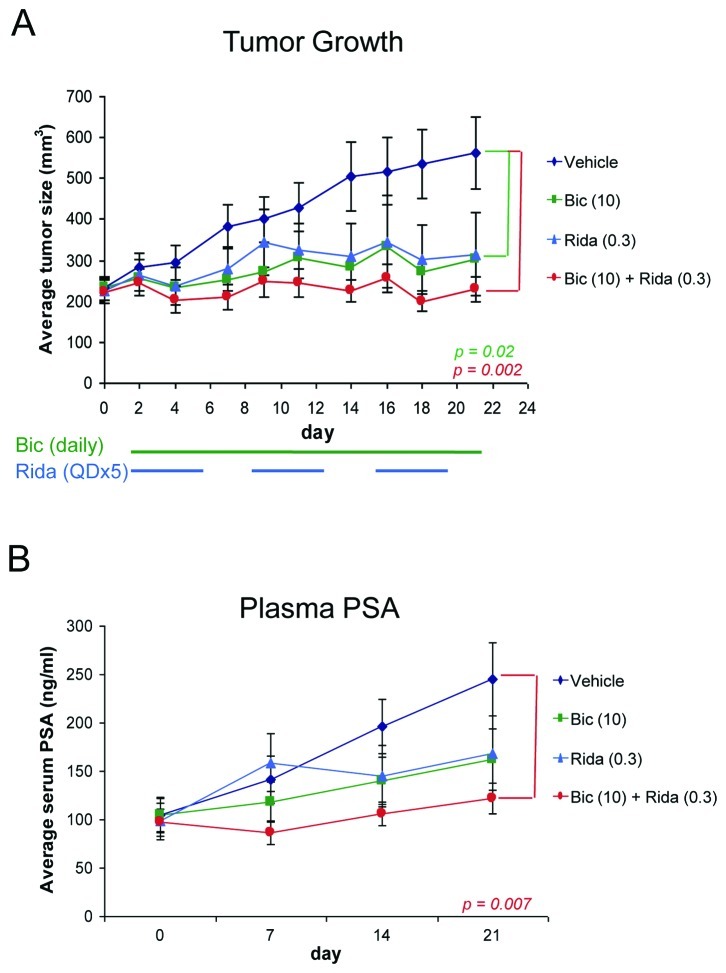
Ridaforolimus and bicalutamide combination induces potent anti-tumor activity with corresponding PSA level reduction *in vivo*. Mice bearing C4-2 prostate cancer xenografts (200 mm^3^) were randomized into four groups (n=10 mice/group). Mice were treated with either vehicle, bicalutamide (Bic; 10 mg/kg), ridaforolimus (Rid; 0.3 mg/kg) or the combination. Ridaforolimus was administered daily for 5 days followed by a 2 day break (QDx5), and bicalutamide administered daily. Three cycles of dosing were completed (21 days). (A) Mean tumor volumes were calculated using caliper measurements and data are plotted as mean ± SE over treatment time. (B) Blood was harvested from the animals on days 0, 7, 14 and 21 and plasma PSA levels determined by ELISA. Data are presented as mean serum concentrations (ng/ml) ± SE for each treatment group.

Finally, we evaluated the combinatorial effect of ridaforolimus and bicalutamide on prostate tumor growth in the C4-2 xenograft model. We used intact nude mice for this study because although C4-2 cells are able to grow tumors in castrated mice, we found that the growth is not robust enough to use for evaluation of efficacy (data not shown). Daily dosing of bicalutamide and a 5 days per week schedule for ridaforolimus were used as this recapitulates the dosing regimens being explored in clinical studies. Single agent ridaforolimus and bicalutamide reduced tumor growth by 73% and 79%, respectively, at defined submaximal doses Fig. 4A). Consistent with the earlier *in vitro* observations, the ridaforolimus-bicalutamide combination exhibited improved and potent antitumor activity, almost completely abrogating tumor growth (TGI = 98%). The combination was also well tolerated, as evidenced by no significant changes in body weight over the course of treatment (data not shown). Importantly, plasma PSA levels were again tightly linked to tumor growth in the combination-treated mice (Fig. 4B), suggesting that PSA may be an accurate and relevant marker of tumor growth in patients undergoing combination therapy.

## Discussion

A number of reports have implicated mTOR signaling as a prominent factor during prostate cancer progression (16,29–31). This can be explained in part by functional loss of PTEN and concomitant activation of the mTOR pathway which is predicted to result in hypersensitivity to mTOR inhibitors (20–22). Consistent with this, we found mTOR signaling to be elevated in PTEN^−/−^ prostate cancer lines relative to PTEN^+/+^ lines, and that PTEN^−/−^ lines exhibit greater sensitivity to ridaforolimus *in vitro*. This suggests PTEN status may predict for sensitivity to ridaforolimus in this tumor type. Several lines of evidence link PTEN inactivation to disease progression and risk of recurrence in prostate cancer (34–38). Moreover in engineered mouse models, loss-of-function of PTEN leads to high grade PIN and/or carcinoma (39,40) and can amplify the tumor-promoting effects of other oncogenes including p53 and p27 (41,42). Taken together, our results identify a potential molecular predictor of response to ridaforolimus treatment in prostate cancer, and also support the possible therapeutic utility of mTOR blockade for treating this disease.

Targeting AR through androgen ablation therapy is the mainstay of prostate cancer treatment. However, these cancers often progress and as a result, treatment options become limited. While often termed ‘androgen-independent’, recent work has shown that the majority of these tumors continue to rely on AR signaling for growth and survival, and several mechanisms have been postulated for reactivation of AR in the castrate environment (3). Overexpression of AR through genomic amplification, as well as mutations that allow activation by reduced androgen levels or by other endogenous steroids, has been observed in recurrent tumors. These cellular alterations are effective in sensitizing the AR pathway since hormone ablation does not completely eliminate serum androgens, with around 10% of baseline testosterone levels still present in castrated men resulting from peripheral conversion in the adrenal glands (43). More recently, ongoing steroidogenesis within prostate tumors and maintenance of intratumoral androgens has been identified as a possible means for these cancers to circumvent low levels of circulating hormones (6). In addition, crosstalk with other growth factor signaling pathways can both stabilize AR and enhance its transcriptional activity. Compensatory regulation between the AR and mTOR pathways has emerged as a key mechanism in the pathogenesis of prostate cancer (10–12). This provides a clear rationale for the investigation of combinatorial strategies using inhibitors of both of these signaling pathways.

We show that simultaneous blockade of both pathways using a combination of ridaforolimus with the antiandrogen bicalutamide resulted in synergistic antiproliferative effects in prostate cancer cell lines, but not in an immortalized normal prostate epithelial line. In normal prostatic epithelia, basal levels of mTOR and AR signaling are each low, as observed in the RWPE-1 cell line. As such, normal tissues would be expected to be relatively unresponsive to treatment with this drug combination. However, in transformed cells, the sustained amplification of both pathways (e.g. through functional PTEN loss) leads to a proliferative circuit and enhanced crosstalk between the two signaling cascades. In this instance, effective blockade of both signaling pathways results in enhanced inhibition of tumor cell proliferation, thus accounting for the strong synergy displayed by ridaforolimus-bicalutamide treatment. This model is supported by recent studies that demonstrate dual AR/mTOR inhibition using antiandrogens with rapamycin or its analogs (12,33,44). In considering mechanism, both androgen deprivation and mTOR inhibition are known to cause cell cycle arrest (45,46). Our data suggest that it is an enhancement of this cytostatic mechanism that accounts for the augmented effects on growth inhibition. In contrast, Wang *et al*(12) have reported that bicalutamide-rapamycin treatment can induce apoptosis in prostate cancer cell lines, although it is not clear whether compound differences, cell lines or duration of treatment account for this discrepancy.

An important finding of this study is that the antiproliferative effects observed following combination treatment *in vitro* translated to potent antitumor activity *in vivo* using a C4-2 xenograft model. This cell line, a derivative of LNCaP, has previously been described as androgen-independent (47,48) and has served as a model for studying growth inhibition in advanced disease. While we have found that C4-2 can grow in the absence of androgens *in vitro* and in castrated mice, our data reveal that these cells do respond to androgen signaling as shown by their sensitivity to bicalutamide treatment in an androgen-rich environment (Fig. 4A). This is supported by previous findings (49) that the endogenous activity of AR in this line exhibits both androgen-inducibility and ligand-independent elements. Although not examined here, additional mechanisms such as intratumoral production of androgens may potentially contribute to the anti-androgen responsiveness of these cells *in vivo*. Single agent blockade of either the AR or mTOR pathway showed comparable levels of tumor response; however the combination treatment regimen (based on the clinical dosing schedules for each) resulted in virtually complete inhibition of tumor growth. Consistent with our observations, it was recently reported that the addition of rapamycin enhanced the efficacy of antiandrogen treatment in a PTEN-null, androgen-dependent transgenic mouse model of prostate cancer (33).

A finding of particular significance was our observation that combination treatment resulted in parallel effects on tumor cell growth and plasma PSA levels in the xenograft model. As single agents, ridaforolimus and bicalutamide exerted opposing effects on AR transcriptional activity *in vitro*. In accordance with other reports of mTOR inhibition (12,44), ridaforolimus treatment of the prostate tumor lines increased expression of PSA, despite its inhibitory effects on cell growth. However, the addition of bicalutamide was sufficient to block the ridaforolimus-induced stimulation of PSA, thereby restoring the correlation between growth inhibition and secreted PSA levels both *in vitro* and *in vivo*. This finding has important clinical implications, as serum PSA levels are used to track tumor burden in prostate cancer patients (50,51). Therefore, the antitumor activity and concomitant reduction of serum PSA exhibited by the combination treatment suggests that PSA would provide a relevant clinical marker of tumor growth in patients treated with this regimen.

In summary, we have shown that the mTOR inhibitor ridaforolimus exhibits robust antiproliferative activity in preclinical models of prostate cancer, alone and in combination with an antiandrogen. The degree of sensitivity is associated with the activation state of the AKT pathway. The addition of bicalutamide results in potent antiproliferative and antitumor activity and represents a potential effective combination strategy for the treatment of prostate cancers. Notably, serum PSA levels reflect the tumor inhibition seen in murine models using this treatment regimen. Taken together, these observations provide strong preclinical support for the exploration of this combination as a novel therapeutic approach in prostate cancer patients, particularly those with loss of the tumor suppressor PTEN.

## Abbreviations:

mTOR: mammalian target of rapamycin
AR: androgen receptor
PTEN: phosphatase and tensin homolog
PI3K/AKT: phosphoinositide 3-kinase/protein kinase B
PSA: prostate specific antigen
PIN: prostate intraepithelial neoplasia
